# Laser Additively Manufactured Iron-Based Biocomposite: Microstructure, Degradation, and *In Vitro* Cell Behavior

**DOI:** 10.3389/fbioe.2021.783821

**Published:** 2021-12-02

**Authors:** Youwen Yang, Guoqing Cai, Mingli Yang, Dongsheng Wang, Shuping Peng, Zhigang Liu, Cijun Shuai

**Affiliations:** ^1^ Institute of Additive Manufacturing, Jiangxi University of Science and Technology, Nanchang, China; ^2^ Key Laboratory of Construction Hydraulic Robots of Anhui Higher Education Institutes, Tongling University, Tongling, China; ^3^ School of Energy and Mechanical Engineering, Jiangxi University of Science and Technology, Nanchang, China; ^4^ NHC Key Laboratory of Carcinogenesis, The Key Laboratory of Carcinogenesis and Cancer Invasion of the Chinese Ministry of Education, School of Basic Medical Science, Central South University, Changsha, China; ^5^ School of Electrical Engineering and Automation, Jiangxi University of Science and Technology, Ganzhou, China; ^6^ State Key Laboratory of High Performance Complex Manufacturing, Central South University, Changsha, China

**Keywords:** laser additive manufacturing, iron bone implant, calcium chloride, degradation properties, cell behavior

## Abstract

A too slow degradation of iron (Fe) limits its orthopedic application. In this study, calcium chloride (CaCl_2_) was incorporated into a Fe-based biocomposite fabricated by laser additive manufacturing, with an aim to accelerate the degradation. It was found that CaCl_2_ with strong water absorptivity improved the hydrophilicity of the Fe matrix and thereby promoted the invasion of corrosive solution. On the other hand, CaCl_2_ could rapidly dissolve once contacting the solution and release massive chloride ion. Interestingly, the local high concentration of chloride ion effectively destroyed the corrosion product layer due to its strong erosion ability. As a result, the corrosion product layer covered on the Fe/CaCl_2_ matrix exhibited an extremely porous structure, thus exhibiting a significantly reduced corrosion resistance. Besides, *in vivo* cell testing proved that the Fe/CaCl_2_ biocomposite also showed favorable cytocompatibility.

## Introduction

In recent years, iron (Fe) has been recognized as a potential bone tissue engineering material owing to its good biocompatibility and mechanical properties (Cheng et al., 2013; Yang et al., 2020a). As compared with the other two biodegradable metals, including magnesium and zinc, Fe possesses relatively high mechanical strength and is more suitable for the repair of load-bearing bone tissue (Li et al., 2018). Fe is an essential micronutrient for the human body and participates in metabolism and various physiological functions, such as hemoglobin synthesis, metabolic enzyme activation, and immunity enhancement (Hermawan et al., 2010; Shuai et al., 2021a). As one kind of biodegradable metal, Fe can be naturally degraded and absorbed in the human body, thus avoiding secondary surgery (Spotorno et al., 2020). However, Fe degrades too slowly, which should hinder the formation and growth of new bone and even cause inflammatory reaction (Yang et al., 2018). The slow degradation is related to the formation of passive film (Sharma and Pandey, 2019). According to the theory of phase-forming film, a dense and well-covered product film could be formed on the Fe matrix as corrosion occurred (Kim et al., 2019). The thin film firmly combined with the Fe matrix could be regarded as an independent solid phase (Wang et al., 2020).

Destroying the passive film of a metallic surface by halogen ion breakdown is an effective way to accelerate the degradation (Yin et al., 2020). Halogen ions, such as chlorine, fluorine, and bromine ions, with strong electronegativity could penetrate into the interface between the metallic matrix and the surface film (Liu et al., 2017; Zhang et al., 2018; Gu et al., 2021). In this case, it would generate lattice expansion and resultant tensile stress at the interface, thus destroying the surface passive film. According to the research of Kong et al. (Kong et al., 2018), halogen ion could cause the relaxation of the passive film structure, so as to achieve the effect of breakdown. Wang et al. (Wang et al., 2014) found that halogen ion could change the film passive structure during the formation of the passive film, thus reducing the protective effect and accelerating the matrix corrosion. Zhou et al. (Zhou et al., 2020) also confirmed that halogen ion destroyed the passive film by potentiodynamic polarization.

In this work, calcium chloride (CaCl_2_), as a halide, was incorporated into Fe-based bone implants fabricated by laser powder bed fusion (LPBF). It was expected that CaCl_2_ would release Cl ion to accelerate the corrosion by attacking the passive film covered on the Fe matrix. Meanwhile, it could also offer Ca ion, one nutrient element that could promote cell proliferation and differentiation (Seol et al., 2014; Chen et al., 2020). LPBF, as additive manufacturing technology, was able to easily fabricate bone tissue engineering scaffold with a complicated pore structure and personalized outer shape, thus meeting the application requirement for different patients (Gao et al., 2021a; Shuai et al., 2021b; Yang et al., 2021). Moreover, it utilized high-energy laser beam as a heat source, which could deal with a wide range of material systems, including metals, ceramics, polymers, and their composites (Pérez-Ruiz et al., 2021; Wang et al., 2021). Herein, the microstructure feature, degradation performance, and corrosion behavior for the Fe/CaCl_2_ composite were systematically studied. In addition, the biocompatibility was also evaluated synthetically to assess its potential application in bone defect repair.

## Material and Methods

### Preparation of the Fe/CaCl_2_ Powder

**FIGURE 1:**
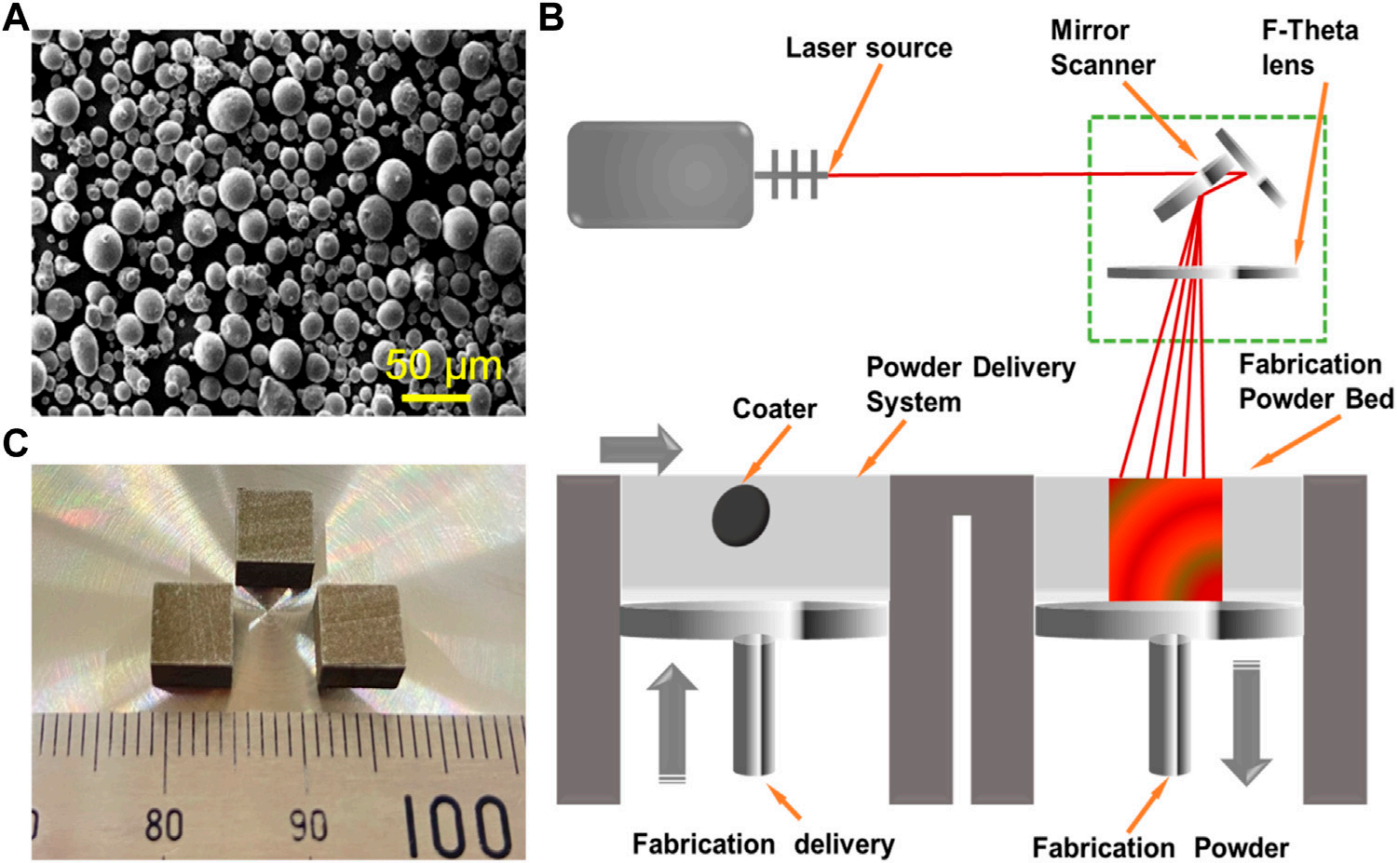
**(A)** Sphere Fe powders. **(B)** Schematic diagram of laser additive manufacturing. **(C)** The as-built parts prepared by LPBF.

Sphere Fe powder (25–50 μm) and CaCl_2_ powder (1–3 μm) were used as raw materials, as shown in Figure 1A. The mixed powder containing 5 wt.% of CaCl_2_ was milled using a miniature planet ball mill (PULVERISETTE 6, Fritsch, Idar-Oberstein, Germany) at room temperature for 2 h. The rotation rate was 200 rpm, and the powder-to-ball weight ratio was determined at 1:10. The ball mill was suspended for half an hour every 15 min to cool down the vial. Before operation, the sealed vial was vacuumed and filled with argon (99.99% purity) aiming to avoid the oxidation. Lastly, the mixture was dried in a vacuum drying oven for 8 h at 125°C.

### LPBF of Fe-Based Biocomposite

The as-milled powder was used for laser additive manufacturing experiments. The manufacturing system consisted of a fiber laser (IPG, 500 W, Germany), a computer control system, and a building chamber filled with purity argon, as displayed in Figure 1B. The optimized process parameters obtained by a series of pre-experiments were as follows: laser power 110 W, scanning rate 12 mm/s, hatching space 50 μm, and layer thickness 50 μm. The laser scanning strategy was set as an alternating scanning strategy, in which the scanning vectors rotated 90° relative to the previous layer. The typical additively manufactured parts are displayed in Figure 1C, and the size was 6 cm in length, 6 cm in width, and 6 cm in height.

### Microstructural Characterization

The as-built parts were grounded to 2,000 mesh utilizing SiC paper and then polished with diamond paste under the lubrication of ethanol. The microstructure was characterized utilizing a scanning electron microscope (SEM, EVO 18, Zeiss, Oberkochen, Germany) at 20 kV. The phase composition was determined utilizing an X-ray diffractometer (XRD, D8 Advance, Karlsruhe, Germany) with Cu-Kα radiation (*λ* = 0.15406 nm) at 40 kV and 40 mA. The scanning range was set at 10°–90°. The texture was investigated using an electron backscattering diffractometer (EBSD, Symmetry, Oxford Instruments, Abingdon, UK), in which the accelerating voltage varied within 12–30 kV by steps of 0.1 kV in a maximum depth range of 4–5 µm. Before EBSD analysis, the samples were prepared by electropolishing in an ethanol solution containing 12 vol.% of water and 8 vol.% of perchloric acid. The surface hydrophilic angles were measured utilizing a contact angle measurement instrument (Shanghai Zhongchen Technology Apparatus Co., Ltd., Shanghai, China) with a 3 ml distilled water droplet suspended from the tip of the microliter syringe.

### Immersion Tests

Immersion tests were implemented to investigate the corrosion behavior. The samples were immersed in self-prepared simulated body fluid (SBF) for 7, 14, and 28 days. SBF mainly contained 142.0 mM of Na^+^, 5.0 mM of K^+^, 1.5 mM of Mg^2+^, 2.5 mM of Ca^2+^, 125.0 mM of Cl^−^, 327.0 mM of HCO^−^, 41.0 mM of HPO^2−^, and 40.5 mM of SO^2−^. After immersion, the samples were washed with distilled water. The corrosion surface was analyzed using SEM. The corrosion product after immersion for 28 days was analyzed utilizing an X-ray photoelectron spectroscope (XPS) with a monochromatic Al Kα radiation. High-resolution spectra were recorded at the pass energy of 12.5 eV with an energy step of 0.1 eV. The film was deeply analyzed by sputtering of argon ion beam with an energy level of 3 keV and a raster of 2 × 2 mm^2^. The sputtering rate was 0.2 nm/s determined on SiO_2_ standard. The samples after immersion 28 days were treated with 200 g/L of CrO_3_ solution to remove the corrosion product. The weight loss was recorded to determine the degradation rate. Besides, the three-dimensional surface morphology was analyzed using an atomic force microscope (AFM, Verco Instruments, USA).

### Electrochemical Experiments

Electrochemical tests were performed using an electrochemical analyzer (PAR model 4,000, Princeton, Oak Ridge, TN, USA) in SBF. The three-electrode battery device was composed of platinum as counterelectrode, saturated calomel as reference electrode, and the testing sample as working electrode. The initial open-loop circuit tests were implemented for 1,800 s to achieve the voltage stability. Tafel polarization curves were obtained at a scanning rate of 1 mV/s. Electrochemical impedance spectroscopy (EIS) curves for samples with different immersing time were measured at a sinusoidal amplitude of 10 mV with the frequency range of 1,000 kHz to 0.01 Hz. The obtained EIS curves were analyzed by ZSimpWin software. The transient time–current curves were measured from −2,000 to −1,000 mV/s at a rate of 1 mV/s. The Mott–Schottky curves were determined at a frequency of 1 kHz to evaluate the semiconductor performance of the passive film.

### *In Vitro* Cell Tests

Cell cytotoxicity was assessed using MG-63 cells (American Type Culture Collection, Rockville, MD, USA). Dulbecco’s modified Eagle’s medium (DMEM) containing 10% fetal bovine serum, 100 units/ml penicillin, and 100 mg/ml streptomycin was applied as culture medium. MG-63 cells were cultured in a 96-well plate containing culture medium for 1 day. Before the experiments, the samples were sterilized using ultraviolet light for 30 min and then immersed in culture medium for 3 days to obtain the extracts. Subsequently, the culture medium was replaced by extracts. After culture for 1, 3, and 5 days, the Calcein AM reagent was used to stain the cells for 15 min. The stained cells were observed utilizing a fluorescence microscope (BX60, Olympus, Tokyo, Japan). Besides, Cell Counting Kit-8 (CCK-8) testing was carried out to assess the cell cytotoxicity. At 1, 3, and 5 days, 10 µl of CCK-8 reagent was dropped into each well and further incubated for 3 h. The absorbance was measured using a microplate reader at 450 nm. Meanwhile, alkaline phosphatase (ALP) staining was carried out to evaluate the differentiation ability of cells cultured for 1 week. After staining, the cells were visualized using a microscope (TE 2000U, Nikon, Tokyo, Japan).

### Statistical Analysis

In the present study, the hydrophilic angle test, immersion tests, electrochemical experiments, and cell tests were carried out at least three times for the averages. SPSS software was used to perform the statistical analysis, in which *p* < 0.05 was recognized to be of statistical difference.

## Results

### Microstructure of Additively Manufactured Biocomposite

**FIGURE 2:**
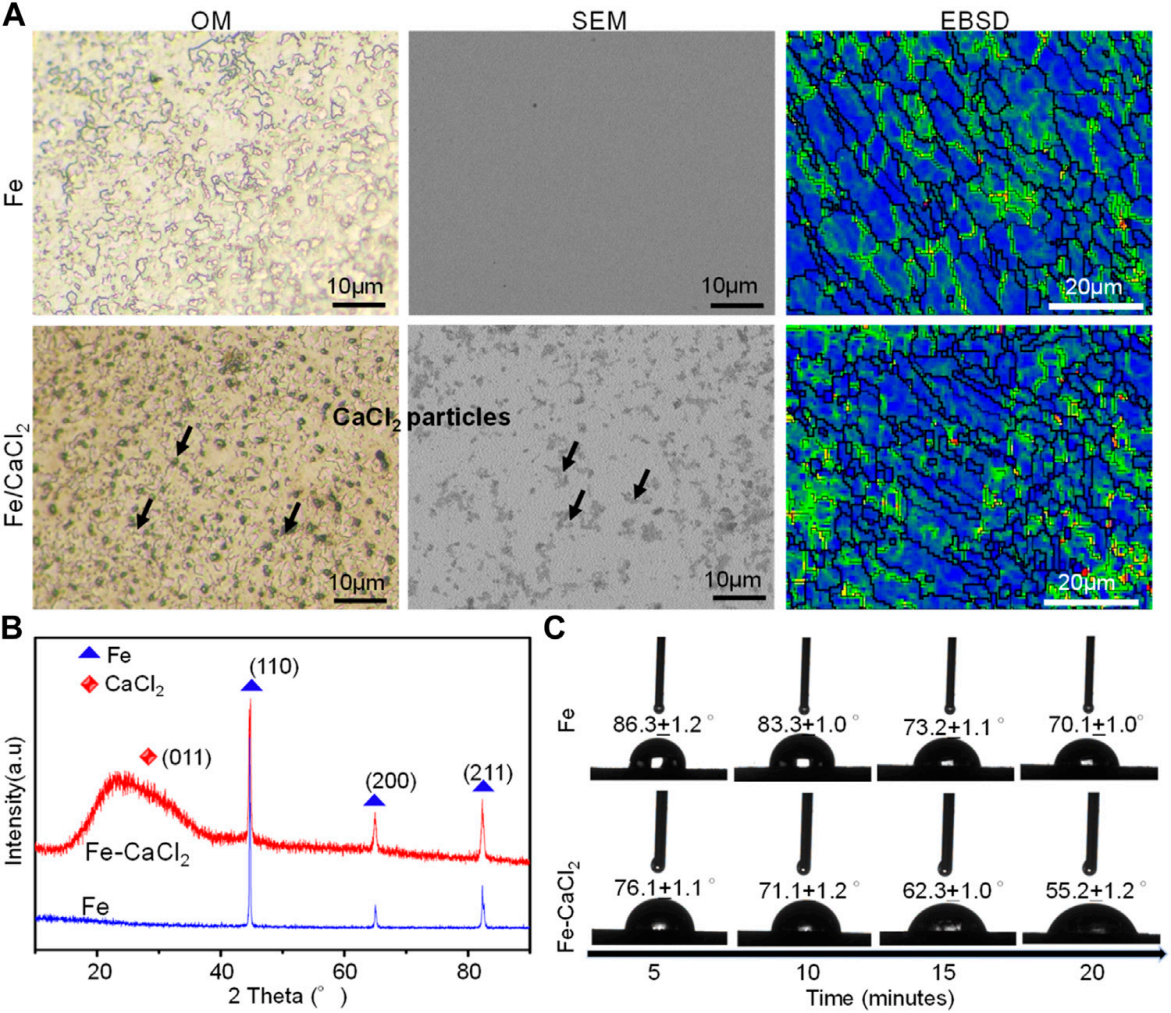
**(A)** The microstructure, **(B)** the XRD spectrum, and **(C)** dynamic results of the hydrophilic angle test.

The microstructure of the Fe and Fe/CaCl_2_ biocomposite after etching is shown in Figure 2A, which clearly revealed the uniform dispersion of CaCl_2_ in the Fe matrix. EBSD mapping further indicated that the Fe/CaCl_2_ composite consisted of refined grains as compared with the Fe part. It was believed that the doped CaCl_2_ pinned at the grain boundary during LPBF and interrupted the crystal growth. The corresponding XRD spectrum of the as-built parts is shown in Figure 2B. A strong peak corresponding to Fe (110) was clearly observed in two samples, whereas a relatively broadened peak corresponding to CaCl_2_ (011) presented in the Fe/CaCl_2_ composite. The hydrophilicity of the Fe and Fe/CaCl_2_ composite was studied using contact angle measurement. As shown in Figure 2C, the water contact angle gradually decreased with contacting time increasing. Encouragingly, the Fe/CaCl_2_ composite showed a significantly decreased water contact angle of 55.2 ± 1.2°, as compared with the Fe part of 70.1 ± 1.0°. It was directly proved that the incorporation of CaCl_2_ improved the surface hydrophilicity, which should be conducive to cell adhesion for the Fe-based biocomposite after implantation (Chen et al., 2018).

### Degradation Behavior in SBF

**FIGURE 3:**
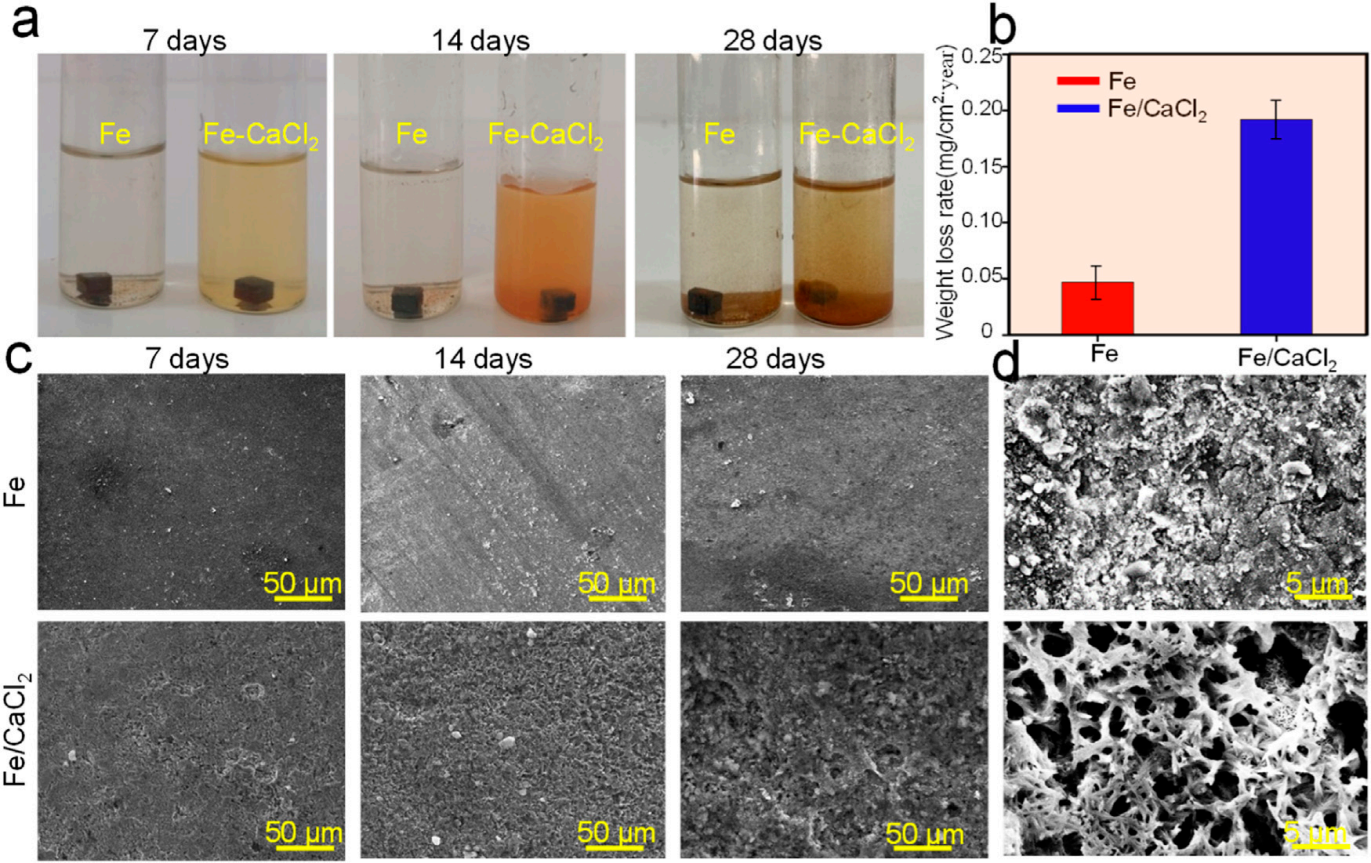
**(A)** The photograph showing the samples immersed in SBF for 7, 14, and 28 days, **(B)** the weight loss rate, **(C)** the corresponding typical corrosion morphology, and **(D)** enlarged view of the corrosion surface.

The degradation behavior of the Fe and Fe/CaCl_2_ biocomposites was evaluated by the SBF immersion test. As shown in Figure 3A, the soaking liquid turned yellow turbid with immersion period gradually extending to 28 days, especially for the Fe/CaCl_2_ composite. This was mainly attributed to the release of Fe^3+^ ion caused by the degradation of the Fe matrix. The corresponding weight losses are displayed in Figure 3B. After immersion for 28 days, the weight loss rates for Fe and Fe-CaCl_2_ were ∼0.05 and 0.18 mg/cm^2^/year, respectively. Additionally, the corrosion surface was observed by SEM, as shown in Figure 3C. The flat corrosion surface with few corrosion products was observed for the Fe part during the whole period. Distinctively, the heavy corrosion product covered on the surface of the Fe/CaCl_2_ composite. It should be noted that the corrosion product layer was characterized with a loosened structure and a lot of corrosion pits on it. These pits obviously became deepened and expanded with immersion time extending. As presented in Figure 3C, an enlarged view of SEM clearly proved the porous structure of the corrosion film for Fe-CaCl_2_ after 28 days’ immersion.

**FIGURE 4:**
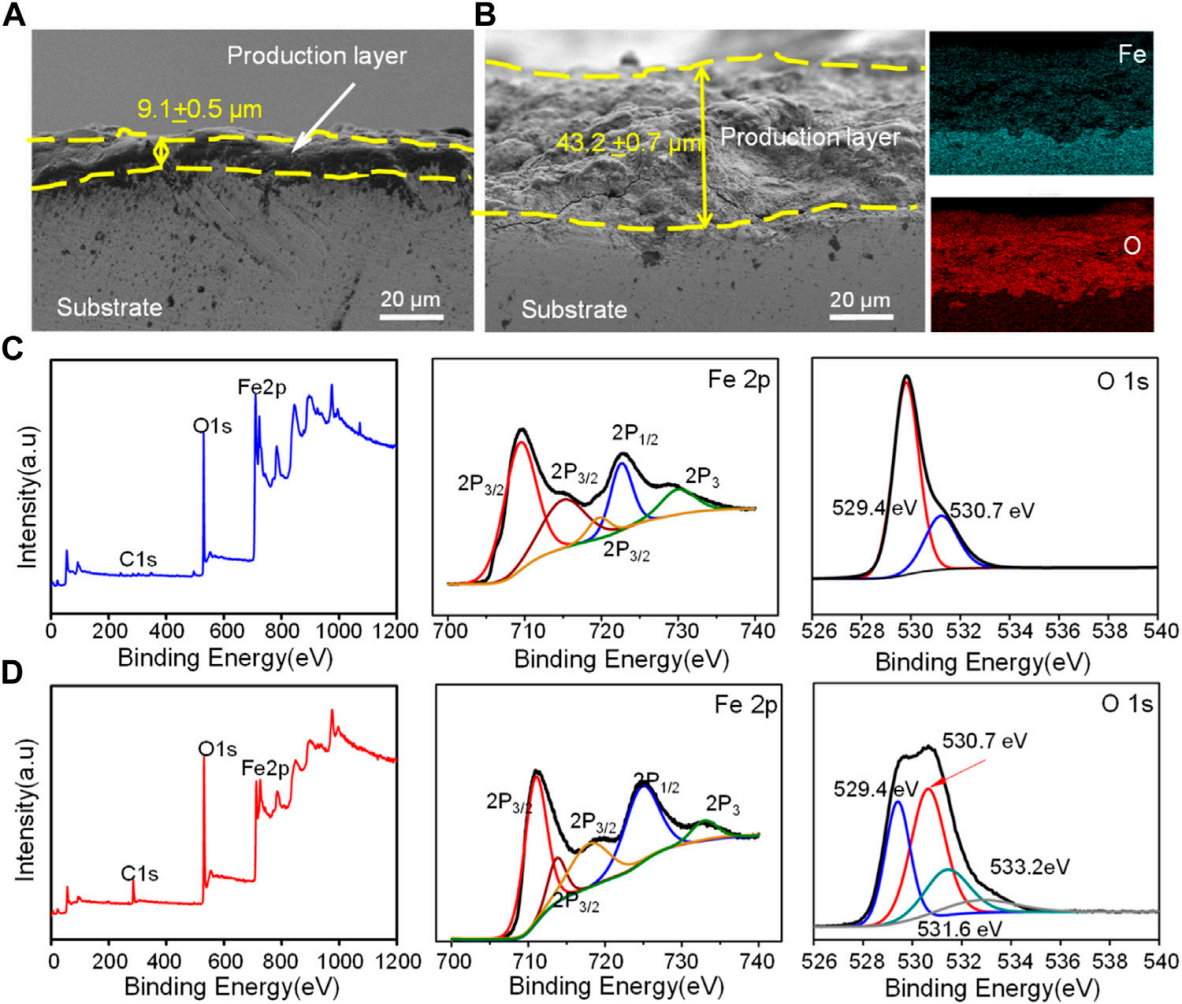
The cross section of corrosion production for **(A)** Fe and **(B)** Fe/CaCl_2_ after immersing for 28 days observed by SEM, and XPS analysis of the oxide layer for **(C)** Fe and **(D)** Fe/CaCl_2_, wherein the sputtering time was 10 s.

The cross section of corrosion production covered on the Fe and Fe/CaCl_2_ composites after immersion for 28 days is shown in Figure 4A. A uniform corrosion product film with a thickness of ∼9.1 μm was observed for the Fe part. For the Fe/CaCl_2_ composite, the thickness of the corrosion film increased to ∼43.2 μm. The element mapping results showed that the corrosion product was mainly composed of Fe and O elements, which was also confirmed by XPS analysis. According to the scanning spectrum, the corresponding high-resolution Fe and O spectra were obtained. Spin-orbit peaks of Fe2p_3/2_ and 2p_1/2_ in the high-resolution Fe spectra are located at 711.4 and 724.6 eV, both of which corresponded to Fe_2_O_3_ as the main corrosion product. The O1s spectrum of the Fe/CaCl_2_ composite in the high-resolution O spectra had two new bands as compared with that of Fe. The peaks of the two bands were located at 531.0 and 529.4 eV, respectively, which were assigned to lattice oxygen and metal oxygen (Wu et al., 2020; Zhou et al., 2020).

**FIGURE 5:**
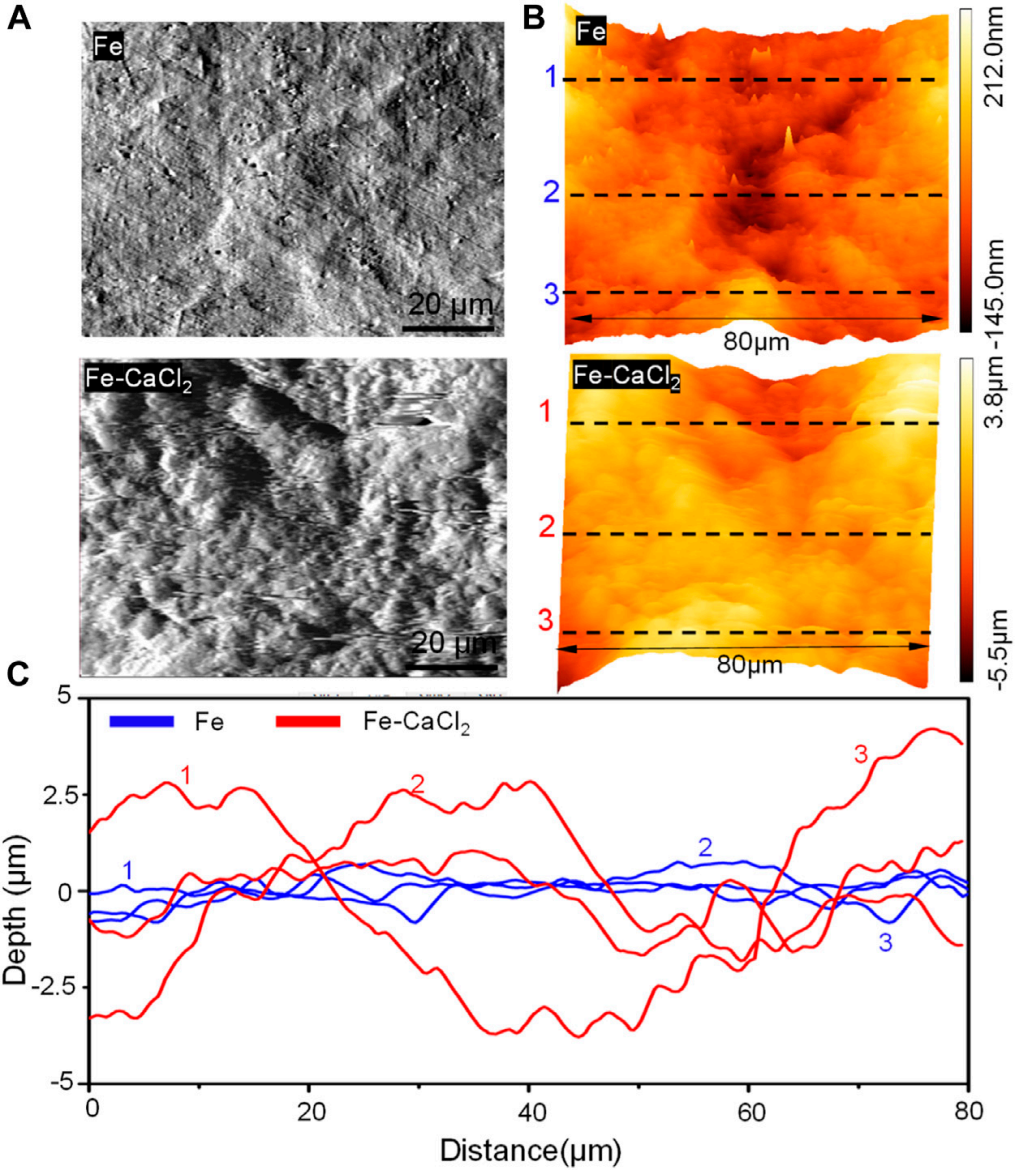
**(A)** The AFM topography of the part surface in SBF for 28 days at 37°C, **(B)** AFM three-dimensional images, and **(C)** the derived surface roughness profiles.

The surface topography of the Fe and Fe/CaCl_2_ composites with the corrosion product removed was analyzed by AFM, as shown in Figure 5. The surface of the Fe matrix showed only a few of small and shallow corrosion pits. In contrast, massive deep and large corrosion pits presented on the Fe/CaCl_2_ composite. As shown in Figure 5B, the three-dimensional AFM images revealed that the surface roughness was only −0.145 to 0.212 μm for the Fe part but enhanced to −5.5 to 3.8 μm for the Fe/CaCl_2_ composite. The curves detected on the Fe/CaCl_2_ composite fluctuated in a larger range than those on the Fe part, as shown in Figure 5C, which also proved that the addition of CaCl_2_ brought about severe pitting corrosion and destroyed the matrix.

### Electrochemical Behavior

**FIGURE 6:**
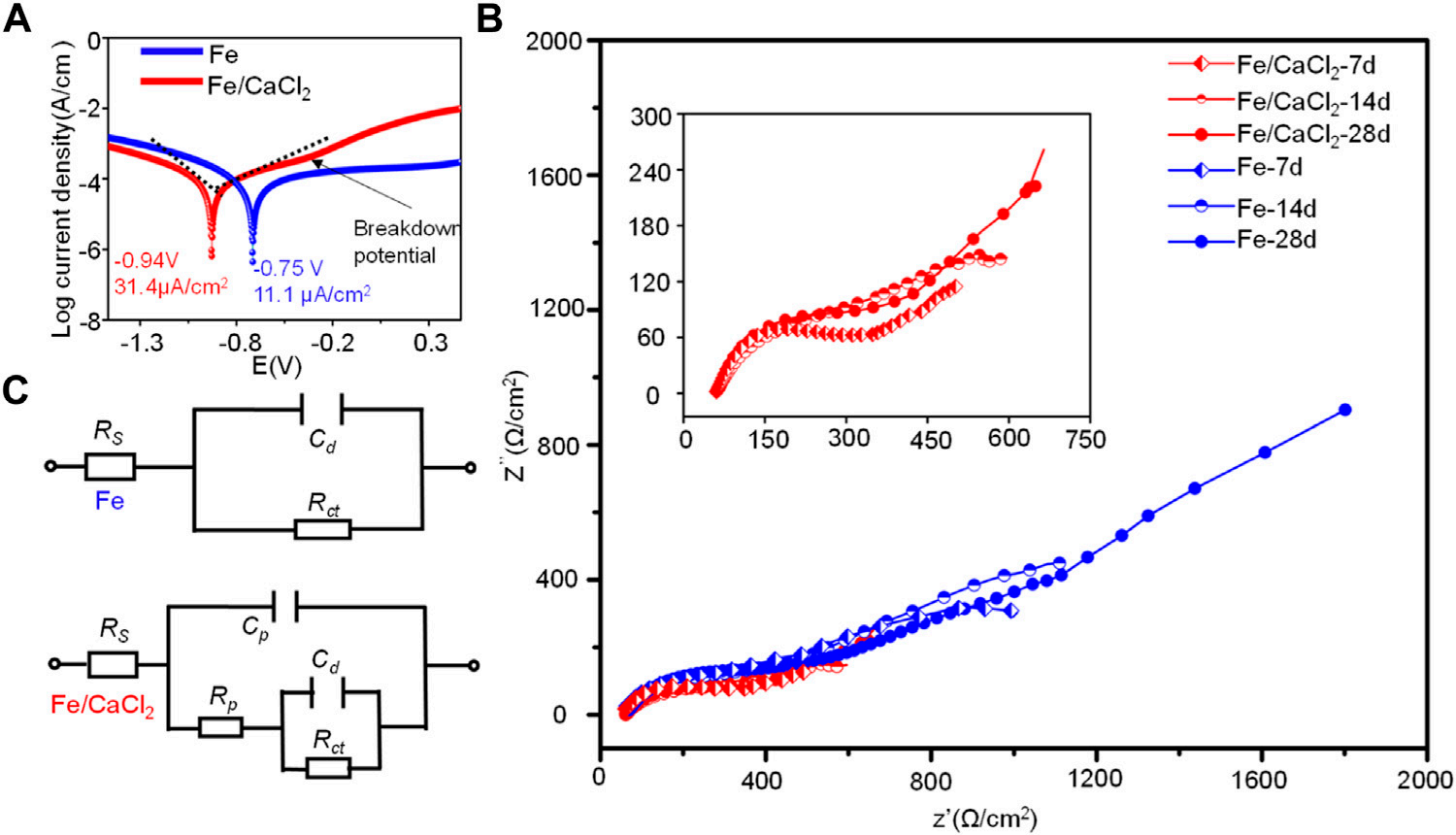
**(A)** Tafel polarization curves for the Fe and Fe/CaCl_2_ composites, **(B)** EIS diagrams, and **(C)** corresponding equivalent circuits after immersing for 7, 14, and 28 days.

The corrosion behavior of the Fe and Fe/CaCl_2_ composites were further investigated by electrochemical tests. As shown in Figure 6A, the potentiodynamic polarization curves showed a typical anodic polarization behavior, resulting in the formation of the passivation film. After doping CaCl_2_, the breakdown potential of the passivation film was negatively shifted. The corrosion potential (E_corr_) and corrosion current density (i_corr_) were obtained by the Tafel region extrapolation method. E_corr_ was −0.75 ± 0.1 V for Fe and −0.94 ± 0.2 V for the Fe/CaCl_2_ composite. However, the i_corr_ of the Fe/CaCl_2_ composite was 31.4 ± 0.9 μA/cm^2^, which was significantly enhanced as compared with that of Fe (11.1 ± 0.4 μA/cm^2^).

The EIS diagrams were obtained after immersion for 7, 14, and 28 days, as shown in Figure 6B. The impedance arcs for the Fe parts gradually increased with immersion time extending from 7 to 28 days, which were closely related with the formation of compact passive film during corrosion. However, the impedance arcs of the Fe/CaCl_2_ composites immersed at 7, 14, and 28 days showed no obvious change and were significantly smaller than that of Fe. It was believed that the passive film was continuously self-destroyed, thus reducing the protection efficiency. The structure of the passive film after immersing for 7, 14, and 28 days was analyzed using equivalent circuits fitted by the EIS spectra, as exhibited in Figure 6C. The circuit for the Fe part indicated the existence of one time constant. It represented a compact passive film which acted as a barrier against corrosion, wherein *R*_ct_ and *C*_d_ were the resistance and capacitance of the passive film, respectively. Distinctively, the circuit for Fe/CaCl_2_ composites showed the existence of two-time constants, which indicated the formation of a porous passive film. In this model, the second time constant was charge transfer reaction, which could be illustrated by the double-layer capacitance *C*_d_ and charge transfer resistance *R*_ct_. The corresponding parameters obtained by fitting the EIS data are shown in Table 1. Obviously, *R*_ct_ increased with immersion time extending for the Fe part, which further proved the high resistance of the product layer and resultant protection efficiency. Meanwhile, its slight increase of *C*_d_ represented a growth of the compact passive film with a long-term stability. As for the Fe/CaCl_2_ composite, a relatively low *R*_ct_ and *C*_d_ indicated its high charge transfer ability and porous product layer with poor protection efficiency (Marinenko and Foley, 1975; Darowicki and Gawel, 2017).

**TABLE 1:** Fitted parameters of EIS data for Fe/CaCl_2_ composite and Fe.

Samples	Fe			Fe/CaCl_2_		
	7 days	14 days	28 days	7 days	14 days	28 days
*R*_ct_ (Ω/cm^2^)	996.3	1,097.8	1,863.2	81.19	103.21	145.34
*C*_d_ (µF)·10^−5^	2.13	2.31	3.45	1.53	1.82	2.01

### *In Vitro* Cell Behavior

**FIGURE 7:**
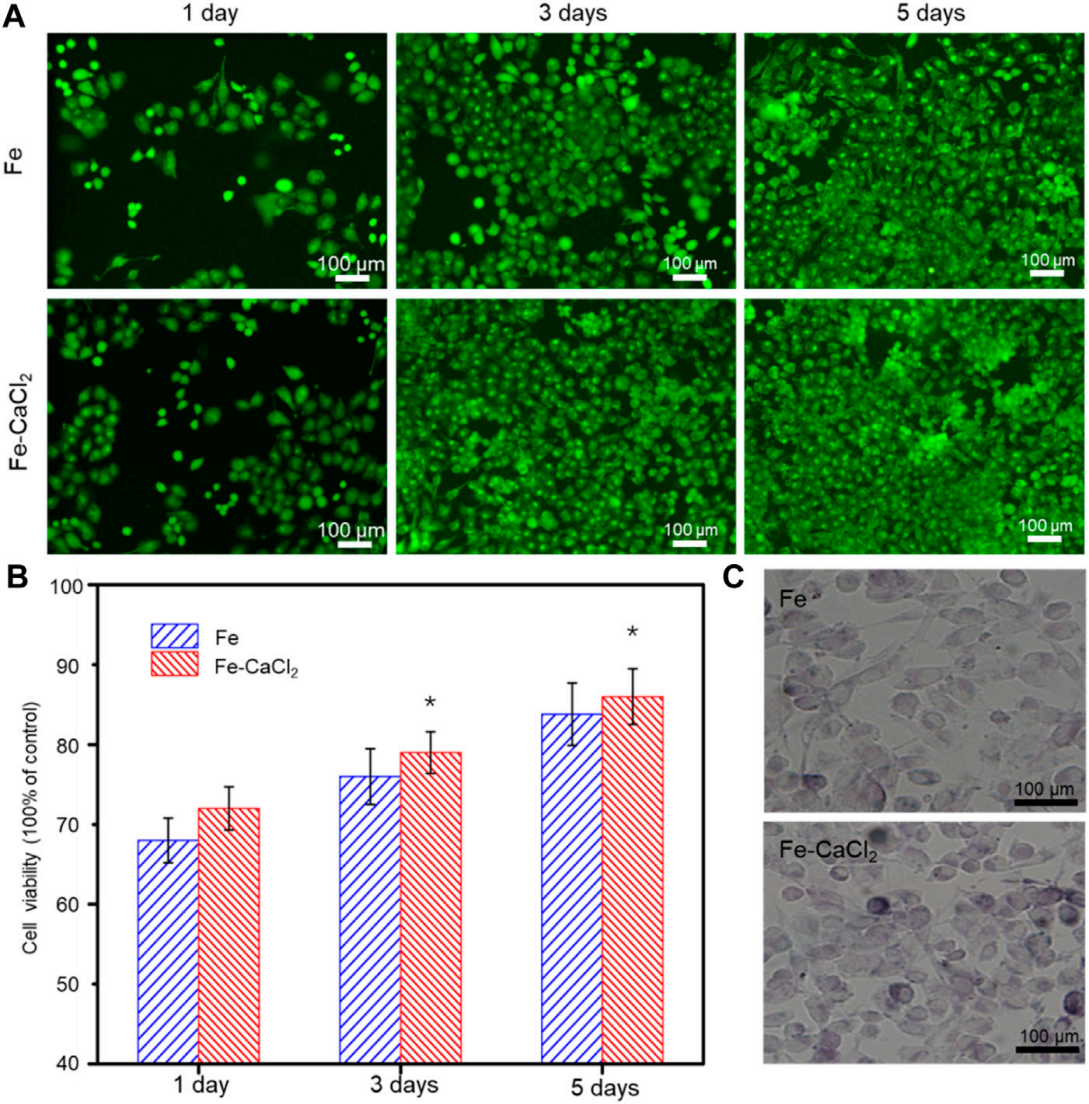
**(A)** The cell fluorescent images after culture for 1, 3, and 5 days, and **(B)** the corresponding 100% concentration extracts using CCK-8 assay. **(C)** ALP stained cell after culture for 7 days.

The biocompatibility of the Fe/CaCl_2_ composite was investigated, with Fe as control. In general, the cells gradually increased with culture time increasing to 5 days, as shown in Figure 7A. In terms of cell morphology, a large number of cells developed pseudopodia and extracellular matrix at day 5. It was indicated that both the Fe/CaCl_2_ composite and Fe exerted no obvious negative effect on cell growth. Furthermore, the cell survival rate was quantitatively analyzed by CCK-8 assay using 100% concentration extracts, as shown in Figure 7B. In both two groups, the cell viabilities were greater than 75%. According to ISO 10993-5-2009, the cytotoxicity was defined as grade 1, which was acceptable for bone implants (ISO, 2009). Notably, the cell viability of the Fe/CaCl_2_ composite group was slightly higher than that of the Fe group. As shown in Figure 7C, the cells cultured in the Fe/CaCl_2_ group showed intensive ALP staining as compared with the Fe group after 7 days’ culture, which revealed the improvement of cell differentiation behavior. It was believed that the released Ca ion, as one of the important components for the human body, might exert a positive effect on cell growth and differentiation.

## Discussion

**FIGURE 8:**
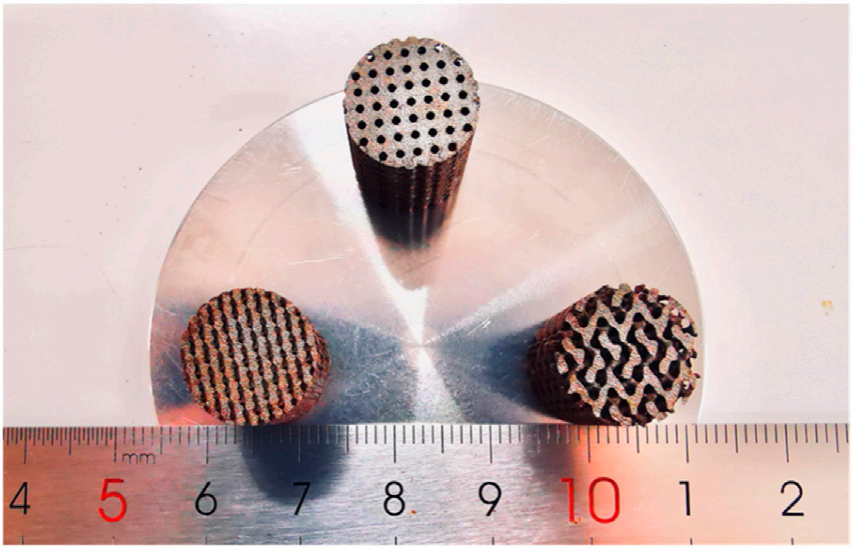
Laser additively manufactured Fe-based scaffolds with porous structure.

Bone substitute was highly demanded aiming to achieve the tissue regeneration and functional reconstruction at critical defect sites (Puleo and Nanci, 1999; Li et al., 2020; Gao et al., 2021b; Qian et al., 2021). In the present study, Fe-based biocomposites were fabricated by laser additive manufacturing. Owing to the unique layer-by-layer fashion, it easily realized the preparation of implant with high geometric complexity and interconnected microporous structure, as well as the customized shape for different patients or defect sites (An and Draughn, 1999). The typical Fe-based scaffolds fabricated by LPBF are presented in Figure 8. Bone tissue engineering scaffolds with different porous structures could be customized according to different needs. The other significant feature of LPBF was its rapid solidification process, which was able to tailor the microstructure (Yang et al., 2020b). Under the effect of laser irradiation, Marangoni convection was generally formed within molten pool, which effectively promoted the rearrangement of reinforcing particles (Kruth et al., 2005; Feng et al., 2021). Subsequently, the molten pool experienced a rapid solidification, namely, the fast-advancing solid–liquid interface, which could refine the grains and promote the uniform dispersion of reinforcing particles by “capture effect,” as shown in Figure 2.

Bone implants also needed to have a degradation rate that matched the bone growth rate, so as to provide space for new bone tissue growth (Trisi et al., 2002). As previous research reported, Fe bone implants degraded too slowly, owing to its high corrosion potential and compact passive film (Cha et al., 2015). In general, a high corrosion potential represented a low corrosion tendency, whereas the compact passive film acted as a barrier that seriously hindered the matrix erosion (Sato and Kudo, 1971). In the present study, CaCl_2_ as a halide was added into Fe bone implants and successfully accelerated the degradation, which was clearly proved by our immersion tests and electrochemical experiments. As shown in Figure 4, massive corrosion products with loose structure covered on the Fe/CaCl_2_ composite. It was believed that the Cl ion released from the matrix resulted in the self-breakdown of the passivation film. In fact, Cl ion possessed strong electronegativity and could attach at the interface between the substrate and the passive film, thereby causing lattice expansion (Zhang et al., 2018). In this case, tensile stress was generally formed at the interface. Under this condition, the passive film located in the convex area of the interface was easily ruptured, thus forming micropores (Gao et al., 2021b). Herein, the Pilling–Bedworth ratio (PBR), defined as the ratio of the volume of oxide (*V*_*ox*_) and volume of metal (*V*_*m*_), was calculated to study the stress state of oxide film. According to the theory of PBR, the perfection or compactness of the oxide film could be expressed by (Zhou et al., 2010):

$$\text{PBR}=\frac{V_{ox}}{V_{m}}=\frac{m_{ox}\cdot \rho_{m}}{n\cdot A\cdot \rho_{ox}}$$ (1)

where *m*_*ox*_, *ρ*_*m*_, and *ρ*_*ox*_ are the molar weight of the oxide, metal, and oxide densities, respectively. *A* and *n* are the atomic weight of the metal and the number of metal atoms in the oxide molecules, respectively. Based on XPS fitting curve analysis (Figures 3B,C), the calculated PBR for the Fe/CaCl_2_ composite had a relatively small value of about 0.93 as compared with Fe (1.92) by using Eq. 1. It was reported that PBR < 1 indicated a discontinuous and porous film with poor protection performance on the matrix (Xu and Gao, 2000; Wang et al., 2019), since it contributed to a smooth way for H_2_O and ions to penetrate into the oxide film. Furthermore, the uncovered Fe matrix would act as anode, whereas the porous passivation film served as cathode of corrosion battery, thus further accelerating the anodic reaction (El-Lateef et al., 2020).

**FIGURE 9:**
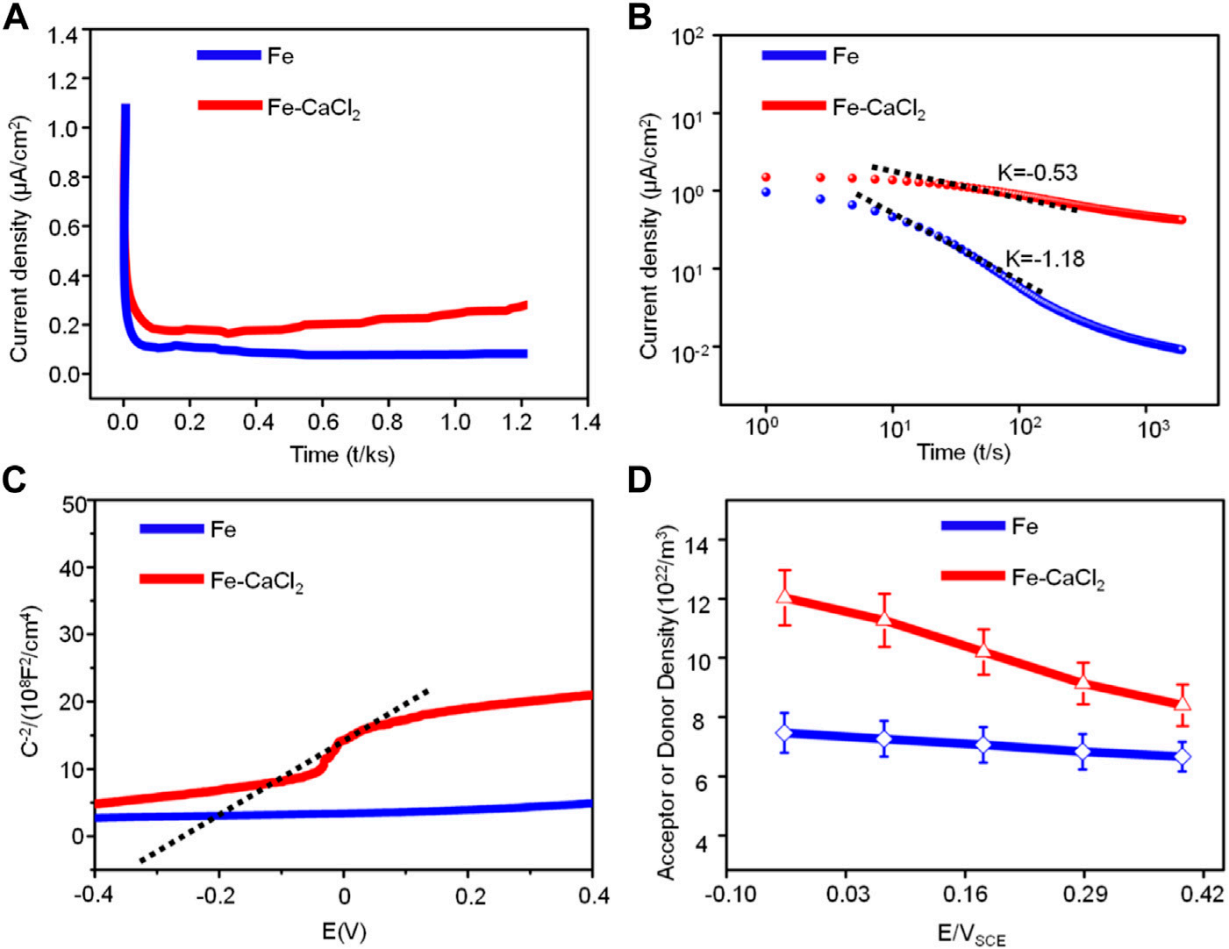
**(A)** The current density–time in linear and **(B)** double logarithmic coordinates plots; **(C)** Mott–Schottky curves of Fe and Fe/CaCl_2_ composite in SBF and **(D)** values of donor density.

On the other hand, our electrochemical experiments also proved that CaCl_2_ reduced the corrosion resistance by means of enhancing the charge transfer ability. Usually, the charge transfer ability was closely related to the structure of the passive film (Liu et al., 2020; Palaniappan et al., 2017). As shown in Figures 9A,B, the typical current density–time in linear and double logarithmic coordinate plots were polarized respectively at the film formation potential of 0.2 V. In the transient, the current density represented the total current resulting from the passive film formation and dissolution in SBF (Kim et al., 2018). As shown in Figure 9A, the initial current density decreased sharply with time extending. It was because the growth of the passive film was relatively fast as compared with the dissolution process (Heo et al., 2016). Subsequently, the current density maintained stably, indicating a balance of the formation and dissolution of the passive film. It was worth noting that the current density for the Fe/CaCl_2_ composite was relatively high as compared with Fe, which corresponded to a weak protection ability of the passive film. Besides, the relatively high level of the current density gradually increased with time extending, which was attributed to the formation of the porous passive film (Lopes et al., 2019). As shown in Figure 9B, the decay rate for the Fe/CaCl_2_ composite was much slow as compared with that of Fe, which further confirmed that the porous passive film improved the charge transfer capability (Bernède et al., 2008).

The electronic property of the passive film was further characterized by *in situ* Mott–Schottky analysis. As shown in Figure 9C, the curve for the Fe/CaCl_2_ composite exhibited a linear region with a positive slope in some potential range, which could be explained in detail by the capacitance of the passive film. According to the Mott–Schottky theory (Taveira et al., 2010), the capacitance (*C*_*sc*_) of passive film could be given by:

$$C_{sc}^{-2}=\frac{2}{\varepsilon \varepsilon_{0}eN_{d}}\left(E-E_{d}-\frac{KT}{e}\right)$$ (2)

where *ε* and *N*_*d*_ are the dielectric constant and donor density of the passive film, respectively. *e*, *E*, and *E*_*d*_ correspond to the electric charge, the potential difference, and the potential in the flat band, respectively. *K*, *ε*_0_, and *T* represent the Boltzmann constant (1.38 × 10^−23^ J/K), the vacuum dielectric constant (8.854 × 10^−12^ F/m), and the thermodynamic temperature in kelvin, respectively. According to Eq. 2, the donor concentration of the passive film increased with the increase in potential difference, as shown in Figure 9D. It was attributed that the movement of oxide anions increased the donor density of the whole corrosion process as passivation started (Windisch et al., 2000). In fact, the high donor density could enhance the sensitivity of the passive film to Cl ion corrosion, which accelerated the penetration and transport of Cl ions to the passive film, thereby resulting in the rapid formation of the porous passive film. In this case, the protective ability of the passive film gradually decreased, which further promoted the destruction of passive film and the corrosion of the Fe matrix (Liu and Wu, 2007; Gerling et al., 2017).

Apart from the suitable porous structure, sufficient mechanical strength, and suitable mechanical properties, bone implants also need to provide a suitable microenvironment for the growth of new tissues (Pooja et al., 2014; Li et al., 2019; Narayanan et al., 2020; Qi et al., 2021). In the present study, the Fe/CaCl_2_ biocomposite easily formed crystalline hydrate with water molecules, thereby showing a significant improvement of wettability, as shown in Figure 2C. Generally, the implants with favorable wettability could quickly promote the adsorption of proteins in body fluids (Russell, 2000; He et al., 2020). As a result, numerous functional groups on proteins were able to bind to cell surface receptors, thus enhancing cell adhesion behavior. Besides, the released Ca^2+^ ion from the Fe/CaCl_2_ biocomposite promoted the cell growth, proliferation, and differentiation, as shown in Figure 7. For degradable implants, bone tissue was able to grow continuously during the process of dynamic degradation, so as to complete the repair of defect tissue and finally realize functional reconstruction (Zhao et al., 2015; He et al., 2021).

## Conclusion

In this study, the Fe/CaCl_2_ biocomposite was fabricated by laser additively manufacturing. The effects of CaCl_2_ on the microstructure, degradation behavior, and *in vitro* cell behavior were investigated. The conclusions were drawn as follows:

1) As compared with Fe, the surface roughness of the Fe/CaCl_2_ composite increased to −5.5 to 3.8 μm after immersion for 28 days. The surface film presented massive porous, and its thickness increased to 43.2 μm.
2) The introduction of CaCl_2_ led to the negative shift of breakdown potential for Fe-based implants and the increase in corrosion current to 31.4 ± 0.9 μA/cm^2^. Besides, the current density and donor concentration of the passive film gradually increased with time, thus resulting in the enhancement of conductivity.
3) The uniformly distributed CaCl_2_ decreased the water contacts of Fe-based bone implants to 55.2 ± 1.2°, which improved the surface hydrophilicity and wettability. Meanwhile, Fe/CaCl_2_ improved the cell growth and differentiation behavior, since the released Ca ion positively affected the cell response.

## Data Availability

The raw data supporting the conclusions of this article will be made available by the authors, without undue reservation.

## References

[B1] An Y. H. Draughn R. A. (1999). Mechanical Testing of Bone and the Bone-Implant Interface. Boca Raton, Florida: CRC Press.

[B2] Bernède J. C. Cattin L. Morsli M. Berredjem Y. Cells S. (2008). Ultra-thin Metal Layer Passivation of the Transparent Conductive Anode in Organic Solar Cells. Solar Energ. Mater. Solar Cell 92 (11), 1508–1515. 10.1016/j.solmat.2008.06.016

[B3] Cha J. Y. Pereira M. D. Smith A. A. Houschyar K. S. Yin X. Mouraret S. (2015). Multiscale Analyses of the Bone-Implant Interface. J. Dent Res. 94 (3), 482–490. 10.1177/0022034514566029 25628271 PMC4814020

[B4] Chen T. Shi P. Zhang J. Li Y. Tian X. Lian J. (2018). Bioinspired Enhancement of Chitosan Nanocomposite Films via Mg-ACC Crystallization, Their Robust, Hydrophobic and Biocompatible. Appl. Surf. Sci. 459, 129–137. 10.1016/j.apsusc.2018.07.194

[B5] Chen Z. Nong Y. Chen Y. Chen J. Yu B. (2020). Study on the Adsorption of OH− and CaOH+ on Fe (100) Surface and Their Effect on Passivation of Steel Bar: Experiments and DFT Modelling. Corrosion Sci. 174, 108804. 10.1016/j.corsci.2020.108804

[B6] Cheng J. Liu B. Wu Y. H. Zheng Y. F. (2013). Comparative *In Vitro* Study on Pure Metals (Fe, Mn, Mg, Zn and W) as Biodegradable Metals. J. Mater. Sci. Tech. 29 (7), 619–627. 10.1016/j.jmst.2013.03.019

[B7] Darowicki K. Gawel L. (2017). Impedance Measurement and Selection of Electrochemical Equivalent Circuit of a Working PEM Fuel Cell Cathode. Electrocatalysis 8 (3), 235–244. 10.1007/s12678-017-0363-0

[B8] El-Lateef H. M. A. Albokheet W. A. Gouda M. (2020). Carboxymethyl Cellulose/metal (Fe, Cu and Ni) Nanocomposites as Non-precious Inhibitors of C-Steel Corrosion in HCl Solutions: Synthesis, Characterization, Electrochemical and Surface Morphology Studies. Cellulose 27 (14), 8039–8057. 10.1007/s10570-020-03292-6

[B9] Feng P. Kong Y. Liu M. Peng S. Shuai C. (2021). Dispersion Strategies for Low-Dimensional Nanomaterials and Their Application in Biopolymer Implants. Mater. Today Nano 15, 100127. 10.1016/j.mtnano.2021.100127

[B10] Gao C. Yao M. Peng S. Tan W. Shuai C. (2021a). Pre-oxidation Induced *In Situ* Interface Strengthening in Biodegradable Zn/nano-SiC Composites Prepared by Selective Laser Melting. J. Adv. Res.. 10.1016/j.jare.2021.09.014 PMC9091777 35572396

[B11] Gao C. Zeng Z. Peng S. Shuai C. (2021b). Magnetostrictive Alloys: Promising Materials for Biomedical Applications. Bioactive Materials. 10.1016/j.bioactmat.2021.06.025 PMC8424514 34541395

[B12] Gerling L. G. Voz C. Alcubilla R. Puigdollers J. (2017). Origin of Passivation in Hole-Selective Transition Metal Oxides for Crystalline Silicon Heterojunction Solar Cells. J. Mater. Res. 32 (2), 260–268. 10.1557/jmr.2016.453

[B13] Gu J.-L. Lu S.-Y. Shao Y. Yao K.-F. (2021). Segregating the Homogeneous Passive Film and Understanding the Passivation Mechanism of Ti-Based Metallic Glasses. Corrosion Sci. 178, 109078. 10.1016/j.corsci.2020.109078

[B14] He F. Lu T. Fang X. Feng S. Feng S. Tian Y. (2020). Novel Extrusion-Microdrilling Approach to Fabricate Calcium Phosphate-Based Bioceramic Scaffolds Enabling Fast Bone Regeneration. ACS Appl. Mater. Inter. 12 (29), 32340–32351. 10.1021/acsami.0c07304 32597161

[B15] He F. Lu T. Feng S. Wang Y. Huang C. Zhang Y. (2021). Alliance of Gallium and Strontium Potently Mediates the Osteoclastic and Osteogenic Activities of β-tricalcium Phosphate Bioceramic Scaffolds. Chem. Eng. J. 412, 128709. 10.1016/j.cej.2021.128709

[B16] Heo J. S. Jo J.-W. Kang J. Jeong C.-Y. Jeong H. Y. Kim S. K. (2016). Water-Mediated Photochemical Treatments for Low-Temperature Passivation of Metal-Oxide Thin-Film Transistors. ACS Appl. Mater. Inter. 8 (16), 10403–10412. 10.1021/acsami.5b12819 27035796

[B17] Hermawan H. Purnama A. Dube D. Couet J. Mantovani D. (2010). Fe-Mn Alloys for Metallic Biodegradable Stents: Degradation and Cell Viability Studies☆. Acta Biomater. 6 (5), 1852–1860. 10.1016/j.actbio.2009.11.025 19941977

[B18] ISO (2009). Biological Evaluation of Medical Devices—Part 5: Tests for *in Vitro* Cytotoxicity. Switzerland: IOS, 10993–10995.‐

[B19] Kim J. Y. Kim A.-Y. Liu G. Woo J.-Y. Kim H. Lee J. K. (2018). Li4SiO4-Based Artificial Passivation Thin Film for Improving Interfacial Stability of Li Metal Anodes. ACS Appl. Mater. Inter. 10 (10), 8692–8701. 10.1021/acsami.7b18997 29461043

[B20] Kim M. Kim G.-H. Lee T. K. Choi I. W. Choi H. W. Jo Y. (2019). Methylammonium Chloride Induces Intermediate Phase Stabilization for Efficient Perovskite Solar Cells. Joule 3 (9), 2179–2192. 10.1016/j.joule.2019.06.014

[B21] Kong D. Dong C. Wei X. Man C. Lei X. Mao F. (2018). Size Matching Effect between Anion Vacancies and Halide Ions in Passive Film Breakdown on Copper. Electrochimica Acta 292, 817–827. 10.1016/j.electacta.2018.10.004

[B22] Kruth J. P. Mercelis P. Van Vaerenbergh J. Froyen L. Rombouts M. J. R. P. J. (2005). Binding Mechanisms in Selective Laser Sintering and Selective Laser Melting.

[B23] Li J. Liu X. Crook J. M. Wallace G. G. (2020). 3D Printing of Cytocompatible Graphene/alginate Scaffolds for Mimetic Tissue Constructs. Front. Bioeng. Biotechnol. 8, 824. 10.3389/fbioe.2020.00824 32766233 PMC7379132

[B24] Li L. Shi J. Zhang K. Yang L. Yu F. Zhu L. (2019). Early Osteointegration Evaluation of Porous Ti6Al4V Scaffolds Designed Based on Triply Periodic Minimal Surface Models. J. Orthopaedic Translation 19, 94–105. 10.1016/j.jot.2019.03.003 PMC6896722 31844617

[B25] Li Y. Jahr H. Lietaert K. Pavanram P. Yilmaz A. Fockaert L. I. (2018). Additively Manufactured Biodegradable Porous Iron. Acta Biomater. 77, 380–393. 10.1016/j.actbio.2018.07.011 29981948

[B26] Liu C. Wu J. J. C. S. (2007). Influence of pH on the Passivation Behavior of 254SMO Stainless Steel in 3. 5% NaCl Solution 49 (5), 2198–2209.

[B27] Liu K. He P. Bai H. Chen J. Dong F. Wang S. (2017). Effects of Dodecyltrimethylammonium Bromide Surfactant on Both Corrosion and Passivation Behaviors of Zinc Electrodes in Alkaline Solution. Mater. Chem. Phys. 199, 73–78. 10.1016/j.matchemphys.2017.06.050

[B28] Liu S. Fang X. Lu B. Yan D. (2020). Wide Range zero-thermal-quenching Ultralong Phosphorescence from Zero-Dimensional Metal Halide Hybrids. Nat. Commun. 11 (1), 4649–9. 10.1038/s41467-020-18482-w 32938942 PMC7494901

[B29] Lopes T. S. Cunha J. M. V. Bose S. Barbosa J. R. S. Borme J. Donzel-Gargand O. (2019). Rear Optical Reflection and Passivation Using a Nanopatterned Metal/dielectric Structure in Thin-Film Solar Cells. IEEE J. Photovoltaics 9 (5), 1421–1427. 10.1109/jphotov.2019.2922323

[B30] Marinenko G. Foley R. T. (1975). Absolute Determination of the Electrochemical Equivalent and the Atomic Weight of Zinc. I. Method, Apparatus, and Preliminary Experiments. J. Res. Natl. Bur. Stan. Sect. A. 79A (6), 737. 10.6028/jres.079a.030 PMC6589413 32184527

[B31] Narayanan N. Jiang C. Wang C. Uzunalli G. Whittern N. Chen D. (2020). Harnessing Fiber Diameter-dependent Effects of Myoblasts toward Biomimetic Scaffold-Based Skeletal Muscle Regeneration. Front. Bioeng. Biotechnol. 8, 203. 10.3389/fbioe.2020.00203 32266234 PMC7105569

[B32] Palaniappan N. Chowhan L. R. Jothi S. Bosco I. G. Cole I. S. (2017). Corrosion Inhibition on Mild Steel by Phosphonium Salts in 1 M HNO 3 Aqueous Medium. Surf. Inter. 6, 237–246. 10.1016/j.surfin.2016.10.003

[B33] Pérez-Ruiz J. D. de Lacalle L. N. L. Urbikain G. Pereira O. Martínez S. Bris J. (2021). On the Relationship between Cutting Forces and Anisotropy Features in the Milling of LPBF Inconel 718 for Near Net Shape Parts. Int. J. Machine Tools Manufacture 170, 103801. 10.1016/j.ijmachtools.2021.103801

[B34] Pooja D. Panyaram S. Kulhari H. Rachamalla S. S. Sistla R. (2014). Xanthan Gum Stabilized Gold Nanoparticles: Characterization, Biocompatibility, Stability and Cytotoxicity. Carbohydr. Polym. 110, 1–9. 10.1016/j.carbpol.2014.03.041 24906721

[B35] Puleo D. A. Nanci A. (1999). Understanding and Controlling the Bone-Implant Interface. Biomaterials 20 (23-24), 2311–2321. 10.1016/s0142-9612(99)00160-x 10614937

[B36] Qi F. Zeng Z. Yao J. Cai W. Zhao Z. Peng S. (2021). Constructing Core-Shell Structured BaTiO3@carbon Boosts Piezoelectric Activity and Cell Response of Polymer Scaffolds. Mater. Sci. Eng. C 126, 112129. 10.1016/j.msec.2021.112129 34082946

[B37] Qian G. Zhang L. Wang G. Zhao Z. Peng S. Shuai C. (2021). 3D Printed Zn-Doped Mesoporous Silica-Incorporated Poly-L-Lactic Acid Scaffolds for Bone Repair. Int. J. Bioprinting 7 (2). 10.18063/ijb.v7i2.346 PMC8114096 33997435

[B38] Russell J. M. (2000). Sodium-potassium-chloride Cotransport. Physiol. Rev. 80, 211–276. 10617769 10.1152/physrev.2000.80.1.211

[B39] Sato N. Kudo K. (1971). Ellipsometry of the Passivation Film on Iron in Neutral Solution. Electrochimica Acta 16 (4), 447–462. 10.1016/0013-4686(71)85182-4

[B40] Seol Y. J. Park J. Y. Jung J. W. Jang J. Girdhari R. Kim S. W. (2014). Improvement of Bone Regeneration Capability of Ceramic Scaffolds by Accelerated Release of Their Calcium Ions. Tissue Eng. Part. A. 20 (21-22), 2840–2849. 10.1089/ten.TEA.2012.0726 24784792 PMC4229868

[B41] Sharma P. Pandey P. M. (2019). Corrosion Behaviour of the Porous Iron Scaffold in Simulated Body Fluid for Biodegradable Implant Application. Mater. Sci. Eng. C 99, 838–852. 10.1016/j.msec.2019.01.114 30889759

[B42] Shuai C. He C. Peng S. Qi F. Wang G. Min A. (2021). Mechanical Alloying of Immiscible Metallic Systems: Process, Microstructure, and Mechanism. Adv. Eng. Mater. 23, 2001098. 10.1002/adem.202001098

[B43] Shuai C. He C. Peng S. Qi F. Wang G. Min A. (2021). Mechanical Alloying of Immiscible Metallic Systems: Process, Microstructure, and Mechanism. Adv. Eng. Mater. 23 (4), 2001098. 10.1002/adem.202001098

[B44] Spotorno R. Ghiara G. Latronico G. Carlini R. Mele P. Artini C. J. J. O. E. M. (2020). Corrosion of the Filled Skutterudite Sm 0.1 (Fe 0.45 Ni 0.55) 4 Sb 12 by NaCl Solutions. Electrochem. Study, 1–9.

[B45] Taveira L. V. Montemor M. F. Da Cunha Belo M. Ferreira M. G. Dick L. F. P. (2010). Influence of Incorporated Mo and Nb on the Mott-Schottky Behaviour of Anodic Films Formed on AISI 304L. Corrosion Sci. 52 (9), 2813–2818. 10.1016/j.corsci.2010.04.021

[B46] Trisi P. Lazzara R. Rao W. Rebaudi A. J. I. J. o. P. Dentistry R. (2002). Bone-implant Contact and Bone Quality: Evaluation of Expected and Actual Bone Contact on Machined and Osseotite Implant Surfaces, 22.6. 12516825

[B47] Wang D. Deng G.-w. Yang Y.-q. Chen J. Wu W.-h. Wang H.-l. (2021). Interface Microstructure and Mechanical Properties of Selective Laser Melted Multilayer Functionally Graded Materials. J. Cent. South. Univ. 28 (4), 1155–1169. 10.1007/s11771-021-4687-9

[B48] Wang H. Yu W. Shen S. (2019). Chemo-mechanical Coupling Effect in the High-Temperature Oxidation of Metal Materials: A Review. Sci. China Technol. Sci. 62 (8), 1246–1254. 10.1007/s11431-018-9500-y

[B49] Wang L. Dou Y. Han S. Wu J. Cui Z. (2020). Influence of Sulfide on the Passivation Behavior and Surface Chemistry of 2507 Super Duplex Stainless Steel in Acidified Artificial Seawater. Appl. Surf. Sci. 504, 144340. 10.1016/j.apsusc.2019.144340

[B50] Wang Z. B. Hu H. X. Liu C. B. Zheng Y. G. (2014). The Effect of Fluoride Ions on the Corrosion Behavior of Pure Titanium in 0.05M Sulfuric Acid. Electrochimica Acta 135, 526–535. 10.1016/j.electacta.2014.05.055

[B51] Windisch C. F. Jr Exarhos G. J. Technology A S. (2000). Mott-schottky Analysis of Thin ZnO Films. J. Vacuum Sci. Tech. A: Vacuum, Surf. Films 18 (4), 1677–1680. 10.1116/1.582406

[B52] Wu P. Zhu X. Xu L. Peng W. Zhao G. (2020). Effect of Stray Current Coupled with Chloride Concentration and Temperature on the Corrosion Resistance of a Steel Passivation Film. Electrochemistry Commun. 118, 106793. 10.1016/j.elecom.2020.106793

[B53] Xu C. Gao W. (2000). Pilling-Bedworth Ratio for Oxidation of Alloys. Mater. Res. Innov. 3 (4), 231–235. 10.1007/s100190050008

[B54] Yang C. Huan Z. Wang X. Wu C. Chang J. (2018). 3D Printed Fe Scaffolds with HA Nanocoating for Bone Regeneration. ACS Biomater. Sci. Eng. 4 (2), 608–616. 10.1021/acsbiomaterials.7b00885 33418749

[B55] Yang H. Jia B. Zhang Z. Qu X. Li G. Lin W. (2020). Alloying Des. biodegradable zinc as promising bone Implants load-bearing Appl. 11 (1), 1–16. 10.1038/s41467-019-14153-7 PMC6972918 31964879

[B56] Yang Y. He C. Dianyu E E. Yang W. Qi F. Xie D. (2020). Design, Mg Bone Implant: Features, Developments and Perspectives. Mater. Des. 185, 108259. 10.1016/j.matdes.2019.108259

[B57] Yang Y. Lu C. Shen L. Zhao Z. Peng S. Shuai C. (2021). *In-situ* Deposition of Apatite Layer to Protect Mg-Based Composite Fabricated via Laser Additive Manufacturing. J. Magnesium Alloys. 10.1016/j.jma.2021.04.009

[B58] Yin X. Wang H. Sun S. Han E.-H. (2020). Comparative Study on the Adsorption Behaviors of O and Cl on Fe(110) Surfaces with Different Cr Content. Mater. Today Commun. 24, 101122. 10.1016/j.mtcomm.2020.101122

[B59] Zhang B. Wang J. Wu B. Guo X. W. Wang Y. J. Chen D. (2018). Unmasking Chloride Attack on the Passive Film of Metals. Nat. Commun. 9 (1), 2559–9. 10.1038/s41467-018-04942-x 29967353 PMC6028649

[B60] Zhao J. Lai H. Lyu Z. Jiang Y. Xie K. Wang X. (2015). Hydrophilic Hierarchical Nitrogen-Doped Carbon Nanocages for Ultrahigh Supercapacitive Performance. Adv. Mater. 27 (23), 3541–3545. 10.1002/adma.201500945 25931030

[B61] Zhou H. Qu J. Cherkaoui M. (2010). Stress-oxidation Interaction in Selective Oxidation of Cr-Fe Alloys. Mech. Mater. 42 (1), 63–71. 10.1016/j.mechmat.2009.09.007

[B62] Zhou S. Yan Q. Tang C. Mao F. Pu J. Macdonald D. D. (2020). Effect of the Chloride on Passivity Breakdown of Al-Zn-Mg alloy. Corrosion Sci. 163, 108254. 10.1016/j.corsci.2019.108254

